# The categorizations of vasculogenic mimicry in clear cell renal cell carcinoma unveil inherent connections with clinical and immune features

**DOI:** 10.3389/fphar.2023.1333507

**Published:** 2023-12-20

**Authors:** Bo Geng, Weiyang Liu, Jinpeng Wang, Wei Zhang, Zhuolun Li, Nan Zhang, Wenbin Hou, Enyang Zhao, Xuedong Li, Bosen You

**Affiliations:** Department of Urology, The Second Affiliated Hospital of Harbin Medical University, Harbin, China

**Keywords:** ccRCC, vasculogenic mimicry, molecular subtypes, tumor immune microenvironment, drug susceptibility

## Abstract

**Background:** Clear cell renal cell carcinoma (ccRCC) stands as the prevailing variant kidney cancer in humans. Unfortunately, patients with disseminated RCC at diagnosis often have a diminished prognosis. Rapid tumor growth necessitates efficient blood supply for oxygen and nutrients, involving the circulation of blood from vessels to tumor tissues, facilitating tumor cell entry into the extracellular matrix. Vasculogenic mimicry (VM) significantly contributes to tumor growth and metastasis. Within this investigation, we identified vasculogenic mimicry-related genes (VMRGs) by analyzing data from 607 cases of kidney renal clear cell carcinoma (KIRC) in The Cancer Genome Atlas (TCGA) and the Gene Expression Omnibus (GEO) database (https://www.ncbi.nlm.nih.gov/geo/). These findings offer insights into ccRCC progression and metastasis.

**Method:** We identified VMRGs-related subtypes using consistent clustering methods. The signature of the VMRGs was created using univariate Cox regression and LASSO Cox regression analyses. To evaluate differences in immune cell infiltration, we employed ssGSEA. Afterwards, we created an innovative risk assessment model, known as the VM index, along with a nomogram to forecast the prognosis of ccRCC. Additionally, we verified the expression of an important gene related to VM, peroxiredoxin 2 (PRDX2), in tissue samples. Furthermore, we assessed the sensitivity to drugs in various groups by utilizing the pRRophetic R package.

**Results:** Significant predictors of survival rates in both high- and low-risk groups of KIRC patients were identified as VMRGs. The independent prognostic factors for RCC were confirmed by both univariate and multivariate Cox regression analyses, validating VMRG risk signatures. Differences were observed in drug sensitivity, immune checkpoint expression, and responses to immune therapy between patients classified into high- and low-VMRG-risk groups. Our nomograms consistently demonstrated precise predictive capabilities. Finally, we experimentally verified PRDX2 expression levels and their impact on prognosis.

**Conclusion:** The signature predicts patient prognosis and therapy response, laying the groundwork for future clinical strategies in treating ccRCC patients.

## Highlights

• Using the TCGA database and GEO database, a prognostic and immunotherapy effectiveness prediction model were developed for patients with ccRCC.

• The distinctive signature holds the potential to function as a valuable instrument to appraise and forecasting the overall survival rate in ccRCC.

• These four genetic factors could enhance the process of clinical decision-making and optimize the individualized treatment for individuals afflicted with ccRCC.

## Introduction

ccRCC is the predominant form of RCC, accounting for the highest occurrence rate (Cotta et al., 2023). It is characterized by increased hypoxia and the upregulation of angiogenesis-related genes (Hsieh et al., 2017; Linehan and Ricketts, 2019). ccRCC represents a solid tumor with extensive vascularization (Aziz et al., 2013). ccRCC has been treated with anti-angiogenic tyrosine kinase inhibitors (TKIs) like sunitinib (Méjean et al., 2018) and pazopanib (Motzer et al., 2013). However, despite the significant improvement in clinical outcomes compared to a placebo, the results have fallen short of expectations. This raises the question of the possibility of an alternative origin for blood and nutrient provisioning. Although there are theories such as epithelial–mesenchymal transition or the development of cancer stem cells, the requirement for blood and nutrient provision remains crucial in order to support the rapid expansion and, ultimately, the significant size of ccRCCs (Singh and Settleman, 2010; Fendler et al., 2020).

VM is depicted as a novel mode of tumor perfusion (Treps et al., 2021). Unlike traditional tumor angiogenesis, VM entails the formation of channels composed of cancer cells (Hendrix et al., 2016; Delgado-Bellido et al., 2017; Xiang et al., 2018). An increasing amount of evidence suggests that TKIs, like sunitinib, could potentially enhance VM formation. This underscores the potent stimulus that compels tumors to actively pursue nutrient supply, even in the face of angiogenesis inhibition (Zhang et al., 2014; Ribatti et al., 2019). Our prior research on VM in ccRCC also furnishes supporting evidence of its role in fostering tumor growth (You et al., 2021; Liu et al., 2022). Hence, VM plays a crucial role in ccRCC progression.

Traditionally, clinical and pathological patient characteristics have been utilized to assess the risk of ccRCC recurrence and predict disease progression (Graham et al., 2018). In recent years, substantial efforts have been dedicated to identifying molecular biomarkers capable of accurately predicting outcomes in ccRCC patients (Cotta et al., 2023). In pursuit of this goal, numerous studies have devised intricate multigene expression profiles (Frew and Moch, 2015). These profiles, whether utilized independently or in conjunction with the conventional stratification system, have demonstrated their capacity to improve the precision of ccRCC prognosis (Rydzanicz et al., 2013; Frew and Moch, 2015; Wang et al., 2022; Zhang et al., 2022). Nevertheless, the collective insights derived from molecular and clinicopathological parameters still do not provide precise prognostications for patient outcomes. Research aimed at discovering novel biomarkers and molecular techniques is essential for advancing ccRCC prognosis and personalizing medical interventions, including exploring specific VM subclusters and their associations with immune characteristics and prognosis.

In this study, we systematically classified ccRCC into distinct VM phenotypes and observed significant differences in prognosis among these subtypes. Additionally, we identified that the high-scoring group exhibited greater potential for immune escape. Furthermore, our constructed prognostic model demonstrated strong predictive capability. Moreover, through experimental validation, we verified PRDX2 expression’s influence on prognosis and its connection to VM.

## Materials and methods

### Data collection

**FIGURE 1:**
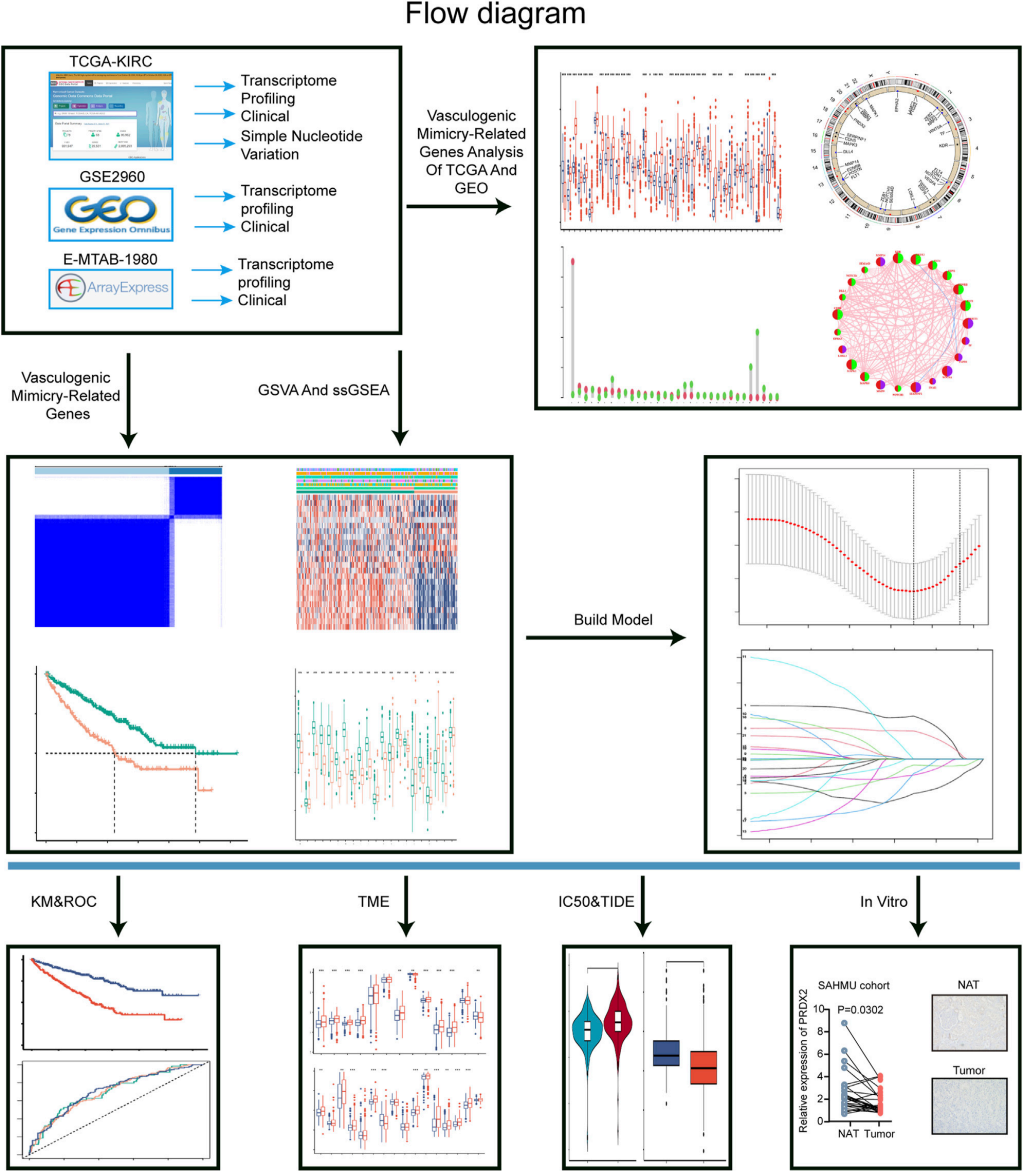
The workflow chart of the whole analysis in this study.

The study was carried out according to the workflow shown in Figure 1. The VMRGs sets (43 genes) were collected from earlier literature research (Luo et al., 2020; Fu et al., 2021; Treps et al., 2021; Wei et al., 2021; Zheng et al., 2021). Data from the TCGA database were downloaded on 25 June 2022 for ccRCC RNA sequencing and clinical characterization (https://portal.gdc.cancer.gov/repository), a dataset of 541 tumor samples and 72 normal tissue samples was included in the study (Liu et al., 2018). Perl (version Strawberry-Perl-5.30.1; https://www.perl.org) was used to extract the RNA-seq data in fragment per kilobase million (FPKM) format. For identical analysis with the E-MTAB-1980 and the GEO data (GSE29609), FPKM values were converted into transcripts per kilobase million (TPM). Following the merging of TCGA and GEO data, we employed the ‘sva’ R package to rectify batch effects, and all data were analyzed using R (version 4.1.3).

### The analysis of consensus clustering for VMRGs GSVA and ssGSEA

To determine the ideal number of subtypes, we performed consensus clustering analysis. Using the ‘GGalluvial’ R package, we analyzed subtypes, overall survival status (OS), and risk scores. Pathway differences between these subtypes were examined through gene set variation analysis (GSVA). Afterwards, we evaluated the infiltration of immune cells across the categories using ssGSEA analysis.

### The construction of a signature based on VMRGs

Initially, we identified predictive VMRGs in the training group by applying a threshold (*p* < 0.05). Then, we utilized LASSO analysis to minimize estimation variance. Afterwards, a predictive signature was identified using multivariate Cox regression analysis. For every ccRCC, we computed a risk score by summing up the products of the gene’s expression value and its corresponding regression coefficient, as per the formula Risk score = ∑N = A, B. n (Coefficient of gene N × Expression value of gene N). Using the median risk score as the threshold, the training cohort was split into groups classified as high-risk and low-risk. For the purpose of assessing the predictive ability of the signature, we created log-rank survival curves and time-dependent receiver operating characteristic (ROC) curves. We also tested the signature’s stability and reliability using similar methods in the E-MTAB-1980 cohorts. The analyses were performed using the R packages ‘glmnet,’ ‘survival,’ and ‘survminer'.

### Evaluation of the forecasting capability of risk indicators

The VMRGs signature’s predictive performance was evaluated using Kaplan-Meier (KM) and Receiver Operating Characteristic (ROC) curve analyses with the assistance of the ‘survival’ and ‘survminer’ R packages. The evaluation of concordance was performed by utilizing the concordance index (C-index). In addition, we performed univariate and multivariate Cox regression analyses to assess the predictive significance of the risk scores. Additionally, we investigated the clustering capability of risk scores using principal component analysis (PCA) and t-SNE (t-distributed stochastic neighbor embedding) analysis. Furthermore, we conducted a comparison between the VMRGs signature and various clinical attributes, including sex, age, and tumor category.

### An illustration of a prognostic nomogram

We evaluated the predictive ability for 1-, 3-, and 5-year OS by employing ROC curves to compute AUC values using time-dependent receiver operating characteristic curves. This analysis took into account risk score, clinical stage, gender, age, and tumor grade. We then constructed a quantitative risk signature for predicting OS rates by creating a nomogram that incorporated the risk score along with other clinical variables. Subsequently, we calibrated the aforementioned nomogram to demonstrate its prognostic value.

### Tumor immune microenvironment characterization with risk score

By employing ssGSEA analysis, the assessment of immune cell infiltration was conducted across different categorizations. The assessment of 47 immunoregulatory checkpoint genes in both cohorts was conducted as the ultimate measure. The Xu et al. website provided gene sets associated with cancer and immune response. The website (http//biocc.hrbmu.edu.cn/TIP/) (Xu et al., 2018) provides additional information. Enrichment scores were precisely computed using the GSVA algorithm to compare gene features related to cancer immune cycles and immunotherapy between two subgroups. *p*-value below 0.05 (Hänzelmann et al., 2013) was considered statistically significant, indicating a significant difference. (Hänzelmann et al., 2013).

### Preprocessing of epigenetic mutation data

The calculation of tumor mutation burden (TMB) involves tallying the number of somatic, coding, base substitution, and insertion-deletion mutations per megabase of the genetic material. Analysis of non-synonymous mutations identifies genetic alterations, whereas those below 5% are classified as code-shifting. TMB was considered high if it exceeded 3. To mitigate statistical prejudice, we eliminated ccRCC individuals who lacked clinical information, gene expression data, or TMB metrics. We employed the ‘maftools’ R package to quantify somatic point mutations in each sample. Somatic changes in ccRCC driver genes were detected in samples exhibiting either low or high-risk scores.

### Significance of the VMRGS in drug sensitivity

The TIDE (Tumor Immune Dysfunction and Rejection) method was utilized to anticipate variations in the responsiveness to immunotherapy among the groups categorized as high-risk and low-risk. To evaluate VMRGS and determine IC50 values for commonly employed chemotherapeutics in ccRCC treatment, the ‘pRRophetic’ R package was employed (Geeleher et al., 2014).

### GeneMANIA

Gene MANIA (http//genemania.org) anticipates genes with similar functions among hub genes and forms a network of protein-protein interactions (PPI) connecting them (Xu et al., 2020). Genes that are functionally similar and genes that are hub genes can also be predicted by it (Franz et al., 2018). The aim of this investigation was to examine functionally comparable genes within hub genes and assess their functional enrichment.

### Cell culture and transfection

The 786-O cell line, which originated from human renal cell carcinoma (RCC), was obtained from the Cell Bank of the Chinese Academy of Sciences. Cells were cultured in RPMI 1640 medium (Gibco, United States) supplemented with 10% fetal bovine serum (FBS) (Gibco, United States) and maintained in an incubator at 37°C with 5% CO2. TRAM2 siRNA and its corresponding si-control were purchased from GenePharma (Shanghai, China). Lipofectamine 3000 reagent (Invitrogen, California, United States of America) was used for cell transfection following the manufacturer’s instructions. After 48 h of transfection, cells were used for protein quantification. The following sequences were employed for targeting PRDX2: 5′-GCC​UGG​CAG​UGA​CAC​GAU​UAA​TT-3' (si-PRDX2-1); 5′-GUG​AAG​CUG​UCG​GAC​UAC​AAA​TT-3' (si-PRDX2-2); 5′-CAG​ACG​CUU​GUC​UGA​GGA​UUA​TT-3' (si-PRDX2-3).

### Sample collection

From 2022 to 2023, all contributors were from the Second Affiliated Hospital of Harbin Medical University, and their specimens were preserved at a temperature of −80°C. Authorization for this investigation was obtained through the approval of the ethics committee at the Second Affiliated Hospital of Harbin Medical University.

### Western blotting analysis

To achieve cell lysis, a cell lysis buffer (Beyotime, China) supplemented with a protease inhibitor cocktail (Seven, China) was used on ice. Afterwards, the cells were collected using cell scrapers from BIOFIL, a company based in China. The Pierce BCA Protein Assay Kit (Beyotime, United States of America) was utilized for protein quantification, with measurements conducted at a wavelength of 562 nm (MD VersaMax, United States of America). Protein samples were applied onto SDS-PAGE gels with varying concentrations, ranging from 7.5% to 12.5%.Following electrophoresis, the proteins underwent transfer onto PVDF membranes (Merck Millipore, United States of America) utilizing an electrophoretic transfer apparatus (Tanon, China). After blocking, The PVDF membranes were subjected to primary antibody incubation for an excess of 12 h at a temperature of 4°C. Afterwards, the protein bands were made visible using chemiluminescence (Tanon) following a 1-h incubation with secondary antibodies under ambient conditions.

### Immunohistochemical staining

IHC staining was conducted following a previously established protocol (Wu et al., 2020). Sections of ccRCC tissue embedded in paraffin were treated with anti-PRDX-2 (1:100, Protein-tech) and observed using a microscope (Leica DM2500P, Germany).

### Matrigel tube formation assay

The *in vitro* assessment of cellular vasculogenic mimicry (VM) capacity was conducted using a Matrigel tube formation assay. In each well of a 96-well plate, 80 μL of Matrigel (at a concentration of 10 mg/mL) was evenly spread and permitted to solidify at 37°C for 1 h. Subsequently, suspended cells (1 × 10^5) were introduced into the culture medium containing varying concentrations of substances within the 96-well plates that had solidified Matrigel. The plates were then incubated in an environment maintained at 37°C with 5% CO2 for a duration of 24 h.

### Extraction of RNA and qRT-PCR

The cells were used to isolate Total RNA with Trizol reagent (Invitrogen, United States of America). Then, cDNA synthesis was performed using the HiScript^®^ III All-in-one RT SuperMix, which is a suitable option for qPCR applications (Vazyme, China). Following that, mRNA quantification was carried out using qRT-PCR assays on the StepOne Plus Real-Time PCR system from Applied Biosystems in the United States. The alterations in mRNA expression were computed utilizing the 2^−ΔΔCT^ technique, while normalizing to β-actin. The PCR primers were specifically synthesized by TSINGKE Biological Technology (located in Nanjing, China) and their details can be found in Supplementary Table S2.

### Statistical analysis

The R programming language was utilized for all statistical computations. Two-sample testing was conducted using the Wilcoxon method. The ‘ggplot2′ and ‘Rtsne’ packages in R were used to perform PCA and t-SNE analyses, respectively. For comparing two independent groups, a *t*-test was applied.

## Results

### Distinctive expression patterns and genetic modifications in VMRGs

**FIGURE 2:**
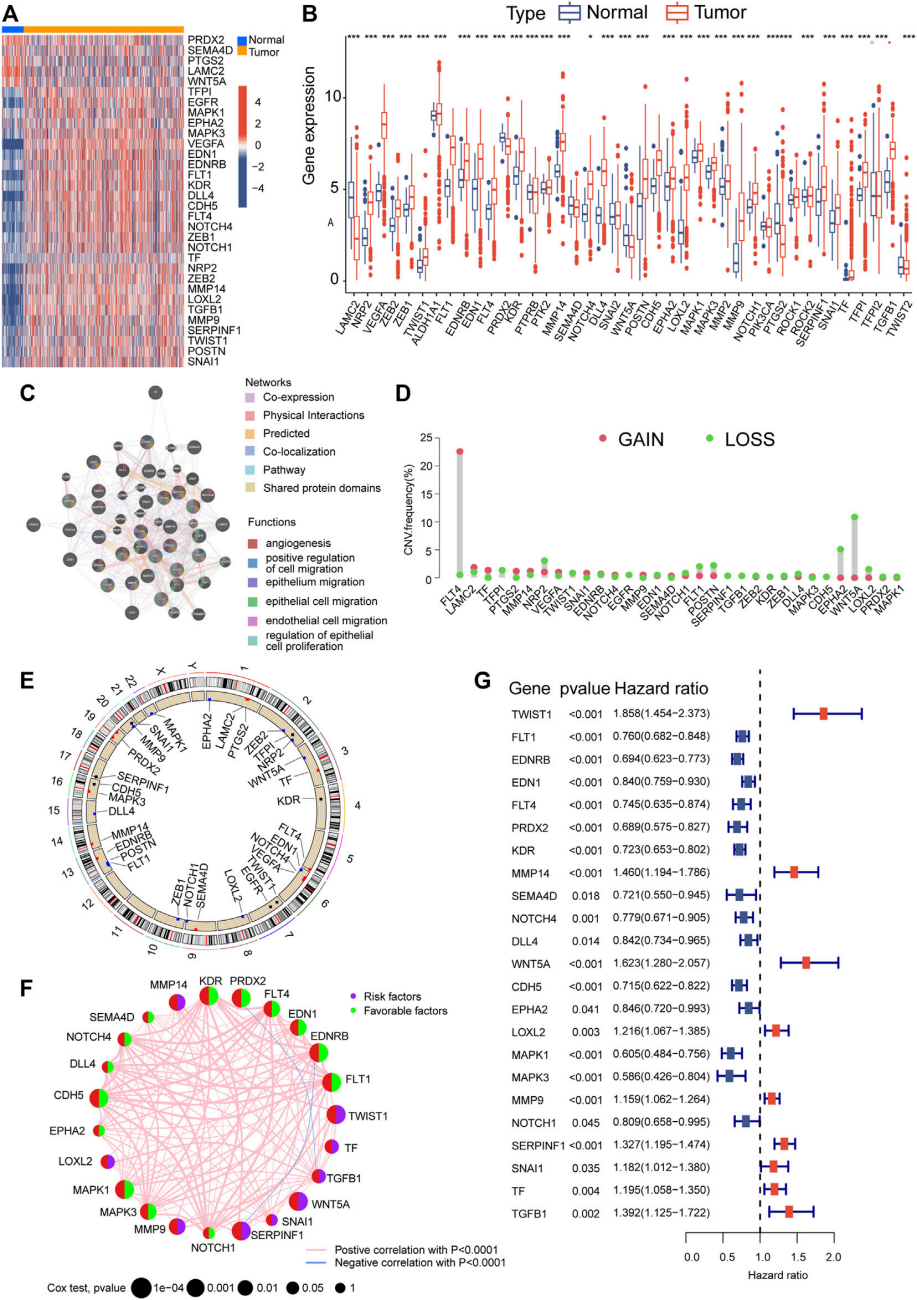
Alterations in gene expression and genetic variations in ccRCC. The TCGA heatmap **(A)** and box plot **(B)** demonstrate that 33 out of the 43 genes associated with VM exhibit noticeably different expression patterns between normal and ccRCC samples. **(C)** GeneMANIA analysis of differentially expressed genes and their co-expressed genes. **(D)** VM regulator CNV values for ccRCC specimens. **(E)** A map of the chromosomal location of CNV alterations in VM regulators. **(F)** Prognostic network depicting VMRGs. **(G)** Forest plot of OS derived from univariate Cox analysis. Significance levels: *p < 0.05, **p < 0.01, ***p < 0.001.

Figure 1 illustrates the overall methodology employed in this study. The compilation of VMRGs, consisting of 43 genes, was derived from prior literature research. The expression profiles of these regulators displayed noteworthy disparities between normal tissues and ccRCC tissues (Figure 2A). Our findings indicate that a majority of genes associated with VM exhibited elevated expression levels in ccRCC. These genes encompass TF, MMP9, VEGFA, LOXL2, DLL4, FLT1, EDN1, NRP2, POSTN, NOTCH4, TGFB1, EGFR, MMP14, KDR, CDH5, FLT4, TWIST1, TFPI, SERPINF1, EDNRB, SNAI1, ZEB1, ZEB2, NOTCH1, MAPK3, EPHA2, and MAPK1. Conversely, SEMA4D, PRDX2, PTGS2, WNT5A, and LAMC2 genes were found to have diminished expression levels (Figure 2B).

The co-expression network analysis confirmed a robust correlation among the co-expression patterns of these genes (Figure 2C). Furthermore, we conducted a copy number variation frequency analysis within the VMRGs. These findings indicate a significant gene dysregulation, with many genes experiencing dysfunction in terms of copy number alterations and deletions. (Figures 2D,E).

By employing univariate Cox analysis, we discovered 23 genes linked to prognosis (*p* < 0.05) within the VMRGs (Figure 2F). Prognostic network maps of VMRGs unveiled ZEB1, FLT1, EDNRB, EDN1, FLT4, PRDX2, KDR, SEMA4D, NOTCH4, DLL4, CDH5, EPHA2, MAPK1, MAPK3, and NOTCH1 as protective factors in ccRCC. Conversely, TWIST1, MMP14, WNT5A, POSTN, LOXL2, MMP9, SERPINF1, and TGFB1 were identified as risk factors (Figure 2G). Prognostic significance of VMRGs in ccRCC patients from the TCGA dataset and GEO (GSE29609) collection was assessed using Kaplan-Meier (KM) and Cox analyses (Supplementary Figure S1). In summary, VM exhibits significant differences in ccRCC and exerts a notably important impact on prognosis.

### Description of phenotypes associated with VM

**FIGURE 3:**
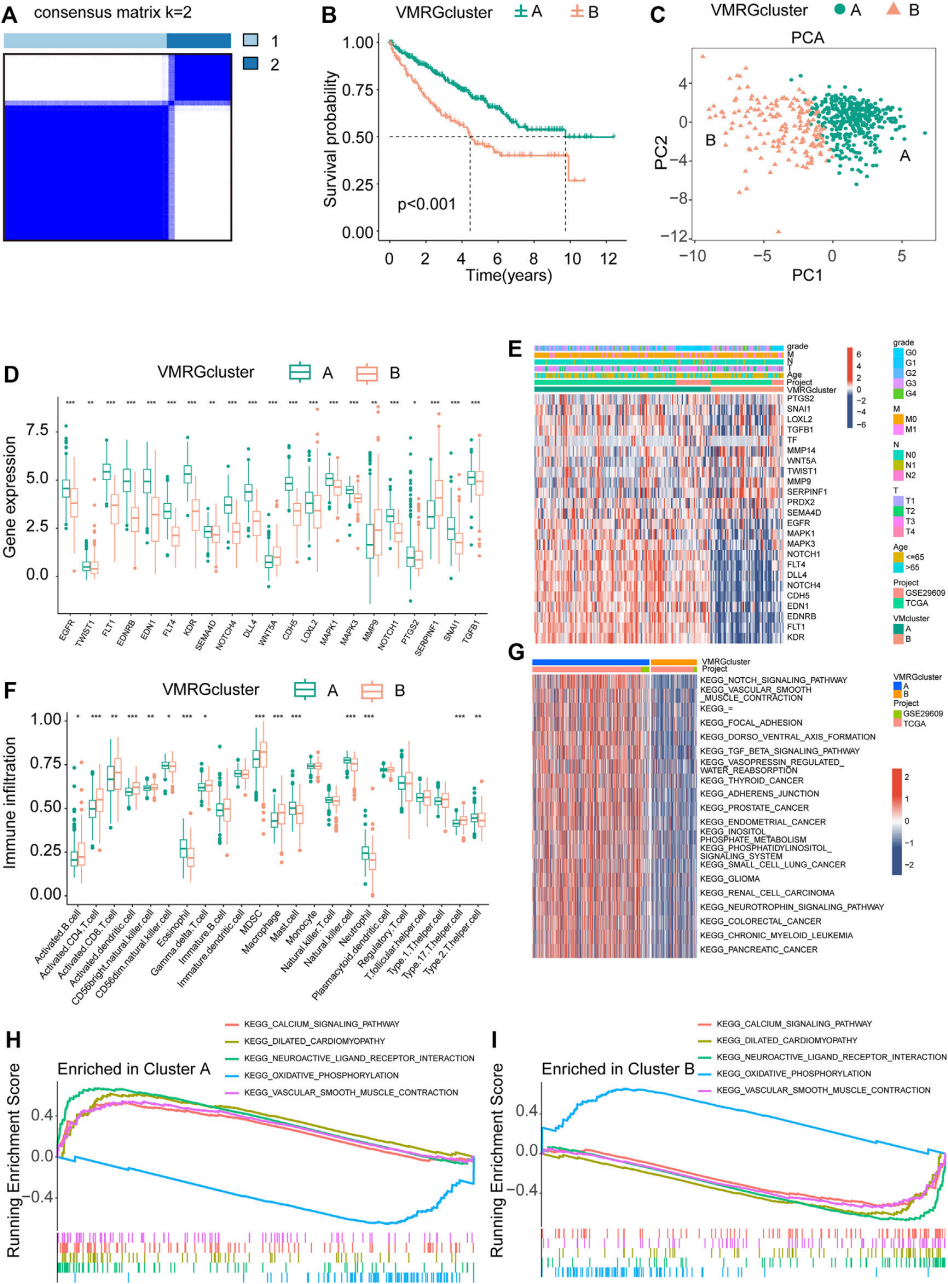
Analysis of signature clusters. **(A, B)** A comparison of the K-M survival curves of the two clusters and the concordance matrix. **(C)** Two clusters were analyzed using PCA. **(D)** The difference between clusters A and B in terms of VMRGs. **(E)** A sophisticated heatmap uncovers clinical associations among the two clusters. **(F)** Conducting differential analyses on two clusters of immune cells and fractions. **(G)** GSVA heatmaps showed the differences in pathways between the clusters. **(H, I)** The GSEA analysis uncovers the signaling pathway connecting the two clusters. Significance levels: *p < 0.05, **p < 0.01, ***p < 0.001.

For this research, we utilized a coherence grid of subcategories to categorize all ccRCC individuals into two main categories, referred to as cluster A and cluster B (Figure 3A). The KM curve, which represents subsequent survival analysis, demonstrated a more positive outlook for ccRCC patients in cluster A compared to cluster B (Figure 3B). By utilizing principal component analysis on the expression profiles of VMRGs, we successfully segregated the data into cluster A and cluster B (Figure 3C). Significantly, we noticed contrasting expression patterns of VMRGs in the two gene clusters, with heightened expression in gene cluster A and diminished expression in gene cluster B (Figure 3D). In order to offer extensive understanding, we combined information from TCGA and GEO (GSE29609) groups, creating a heatmap based on clusters that displays the distribution of age and clinical stage among ccRCC patients (Figure 3E). We utilized the ssGSEA method to appraise the fractions of 23 distinct immune cell types within the two ccRCC clusters. Remarkably, 15 immunocyte types exhibited notable variances between these ccRCC clusters, as depicted in the figure (Figure 3F). Concurrently, GSEA revealed significant distinctions in the top 20 pathways between clusters A and B. Key pathways included NOTCH SIGNALING, AXON GUIDANCE, DORSO VENTRAL AXIS FORMATION, VASOPRESSIN REGULATED WATER REABSORPTION, and various cancer-related pathways (Figure 3G). Additionally, GSEA enrichment analysis intimated that cluster A was significantly augmented in the CALCIUM signaling pathway and VASCULAR SMOOTH MUSCLE CONTRACTION pathway, while the cluster B primarily exhibited enrichment in OXIDATIVE PHOSPHORYLATION pathways (Figure Figure3 H, I). These observations underscore a strong correlation between VM, neurological and vascular function, and tumor development. These results highlight the strong correlation between defined ccRCC clusters based on VMRGs and both prognosis and the immune status of ccRCC patients.

### Developing and validating risk models

**FIGURE 4:**
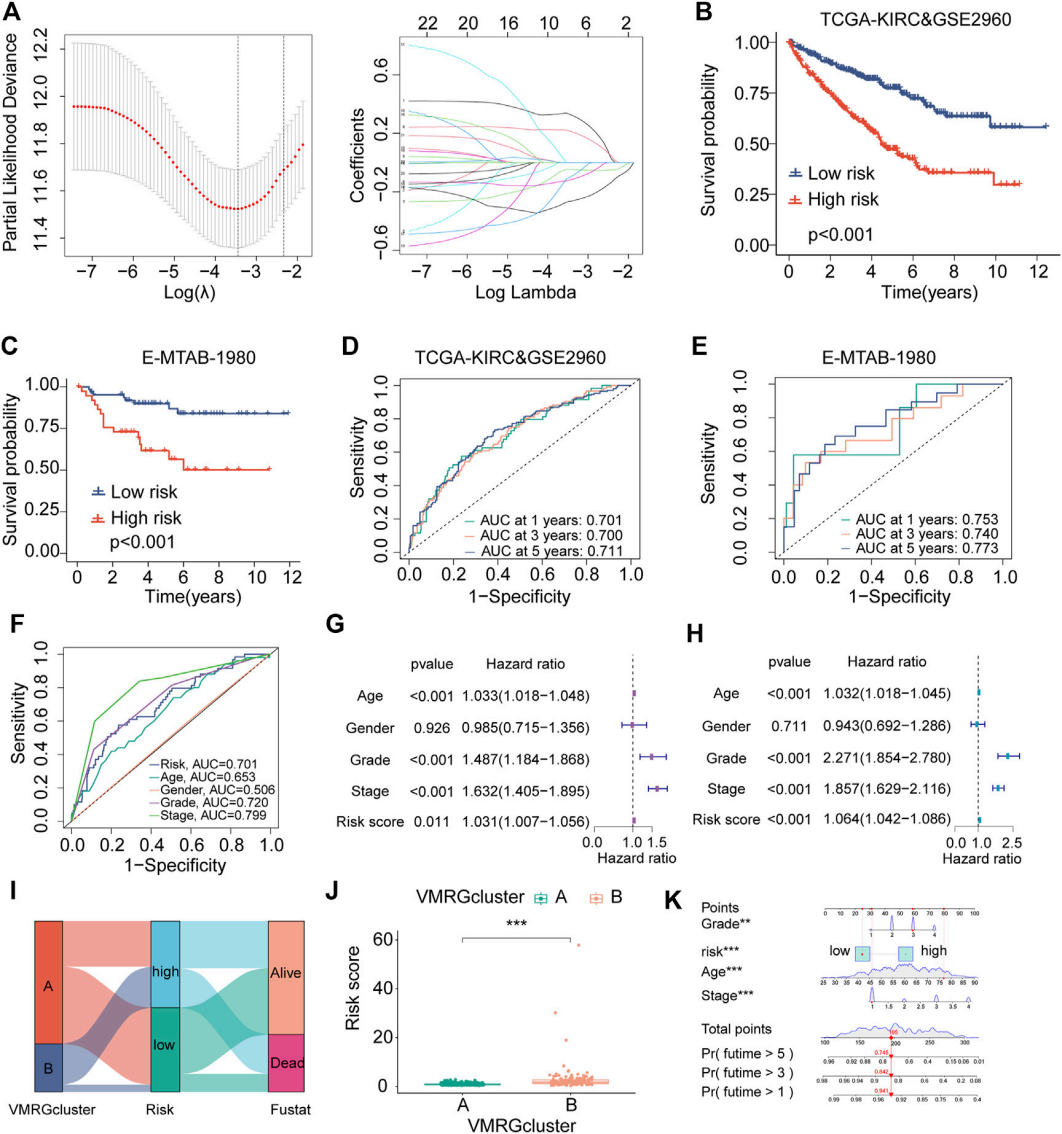
The establishment and validation of risk signatures linked to VM. **(A)** The merged dataset revealed four genes associated with VM that are most closely associated with OS using Lasso Cox analysis. **(B, C)** The Kaplan–Meier analysis of combined dataset and E-MTAB-1980 assess the prognostic importance of the risk model. The constructed model in the combined datasets **(D)** and E-MTAB-1980 **(E)** displays a time-dependent ROC curve. **(F)** ROC curves for risk score, age, gender, grade stage, and classification in the merged datasets. **(G, H)** Multivariate and univariate Cox regression findings in the combined dataset. **(I)** A prognostic model is depicted in a Ggalluvial plot. **(J)** Boxplots representing VM clusters illustrate disparities in risk scores. **(K)** A nomogram amalgamating the risk score of risk scores and clinical variables across the datasets, encompassing age, gender, and stage. Significance levels: *p < 0.05, **p < 0.01, ***p < 0.001.

With the aid of the LASSO regression analysis, four genes were identified as optimal candidates for the model (Figure 4A), which are listed in Supplementary Table S1. Using Kaplan-Meier analysis, it was ascertained that a substantial correlation existed between high-risk scores and adverse outcomes in the merged cohorts (Figure 4B). This finding was corroborated by the E-MTAB-1980 dataset (Figure 4C). Further, survival status, risk score, and risk gene expression distributions were compared between the Merged and E-MTAB-1980 datasets (Supplementary Figure S2A, B).

The prediction model’s effectiveness was assessed using time-dependent ROC curves. The AUCs of the combined dataset at 1, 3, and 5-year intervals were 0.701, 0.700, and 0.711, respectively (Figure 4D). At 1, 3, and 5-year intervals, the time-dependent ROC curves of the E-MTAB-1980 dataset produce area values of 0.753, 0.750, and 0.777, respectively (Figure 4E). ROC curves comparing risk scores with other clinical characteristics (Figure 4F) demonstrated the model’s high predictability.

Risk scores were discerned as significant independent prognostic factors in the assessment of prognostic accuracy through univariate and multivariate Cox regression analyses (Figures 4G,H). The Sankey diagram was used to show the construction of the prognostic model, while the box plots were used to show the risk scores among VM clusters based on how the model was developed. (Figures 4I,J).

A nomogram was created using VMRGs to predict the 1, 3, and 5-year OS in ccRCC patients. This nomogram includes stage, age, grade, and risk score (Figure 4K). The calibration curve demonstrated a close alignment between the observed and predicted outcomes (Supplementary Figure S2C). ROC analyses demonstrated the nomogram’s higher sensitivity as a method for predicting the survival time of ccRCC patients after 1, 3, and 5 years (Supplementary Figure S2D). In summary, the results of this study demonstrate the prognostic accuracy of the proposed model in predicting the outcomes of patients with ccRCC in the future.

### Clinical appraisal of risk model

In order to clarify the connection between the model and clinical traits in the validation group, we utilized Wilcoxon signed-rank tests to examine the correlation between risk groups and pertinent factors. The findings from our study suggest that the risk scores obtained from the model did not show any significant connections with the age of the patients (*p* > 0.05) (Supplementary Figure S3A) and the N stage (*p* > 0.05) (Supplementary Figure S3E). Significant associations with risk were observed for Gender (*p* < 0.05) (Supplementary Figure S3B), tumor grade (*p* < 0.05) (Supplementary Figure S3C), T stage (*p* < 0.05) (Supplementary Figure S3D), and M stage (*p* < 0.05) (Supplementary Figure S3F). The results indicate that the risk model we created is mainly associated with the spread of ccRCC.

### Differences in the immune cell infiltration

**FIGURE 5:**
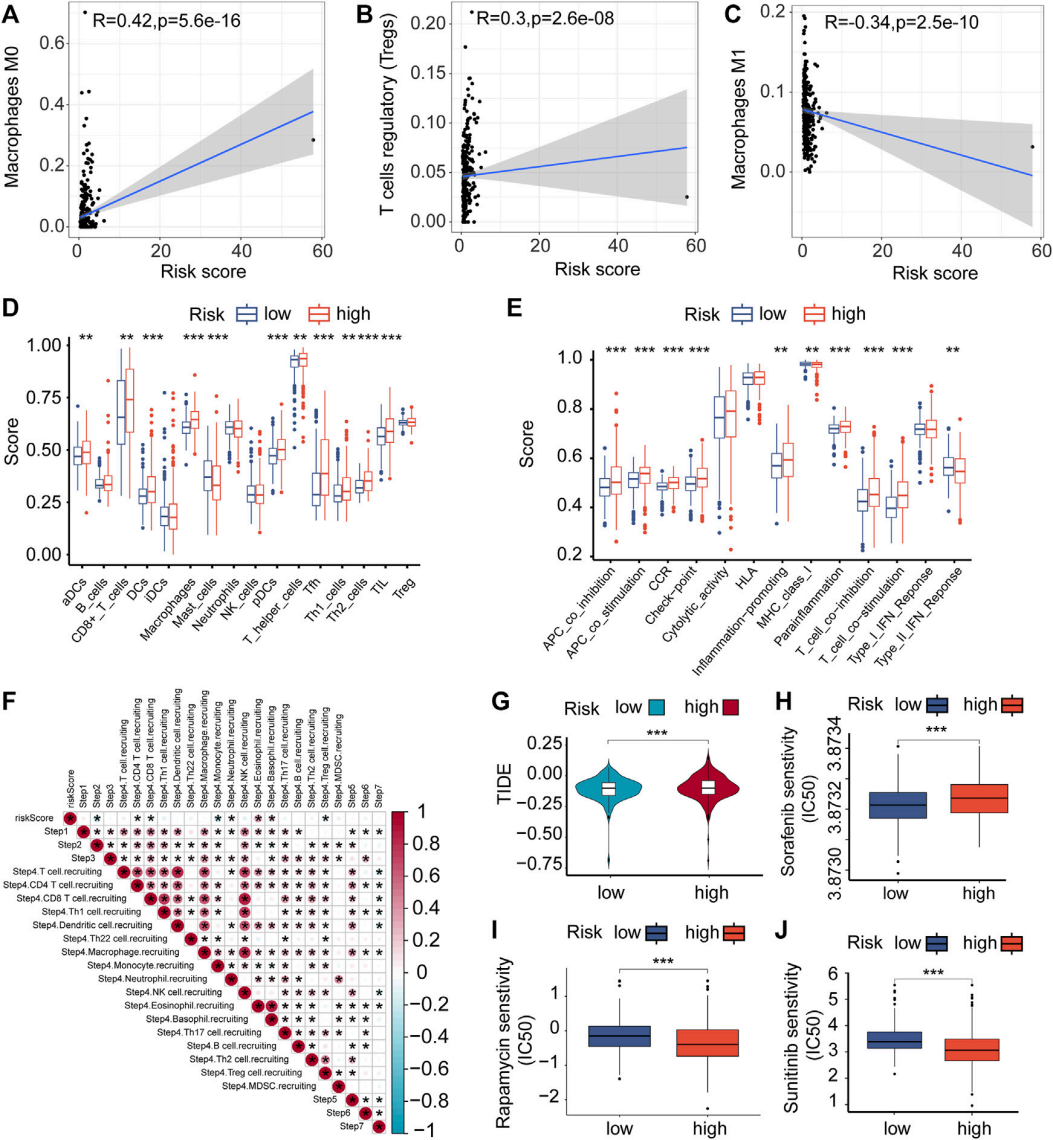
Immune cells infiltrating tumors and chemotherapy. **(A–C)** The relationship between the risk score of VMRGS and the infiltration of immune cells in ccRCC. **(D–E)** ssGSEA scores for infiltration of immune cells **(D)** and the functionality of the immune system **(E)**. **(F)** The interrelation amid the stages of the tumor-immune cycle and the scores indicating risk. **(G)** TIDE scores with low and high risk. Comparing the IC50 levels of Sorafenib **(H)**, Sunitinib **(I)**, and Rapamycin **(J)** in two different prognostic risk groups. Significance levels: *p < 0.05, **p < 0.01, ***p < 0.001.

A Spearman correlation analysis was performed, which showed a noteworthy positive correlation between risk scores and both macrophage M0 and regulatory T cells (Tregs) (Figures 5A,B). In contrast, there was an inverse relationship between Macrophages M1 and risk scores (Figure 5C). Furthermore, notable disparities were observed in the ratios of immune cells and the scores associated with immune functions among the two groups (Figures 5D,E). As a result, a study was conducted to analyze differences in tumor immune pathway functions between the high-risk and low-risk categories (Figure 5F). The results showed heightened activity in specific stages of the process, like Neutrophil attracting (step 4), Eosinophil attracting (step 4), and Basophil attracting (step 4) in the high-risk category, whereas Monocyte attracting (step 4) displayed decreased activity. Significant variations in immune and ESTIMATE scores were evident between the high-risk and low-risk groups, as shown in Supplementary Figure S4A. Furthermore, the group at high risk exhibited an increased expression of PD-1 and CTLA4, which serve as potential targets for checkpoint immunotherapy (Supplementary Figure S4B). The variances in immune reactions among the two categories could be incorporated into antitumor immunotherapy for ccRCC (Figure 5G).

The pRRophetic algorithm was utilized to evaluate the correlation between risk scores and the sensitivity of three frequently prescribed medications (Sunitinib, Rapamycin, and Sorafenib) by determining the half-maximal inhibitory concentration (IC50) in ccRCC. The findings indicated that individuals classified as high-risk displayed greater responsiveness to Sorafenib (Figure 5H), while those classified as low-risk exhibited increased sensitivity to Sunitinib and Rapamycin (Figures 5I, J). The results indicate possible connections between VM, the immune environment, evasion of the immune system, and variations in prognosis among ccRCC patients in the two risk categories.

### Tumor mutational burden analysis

**FIGURE 6:**
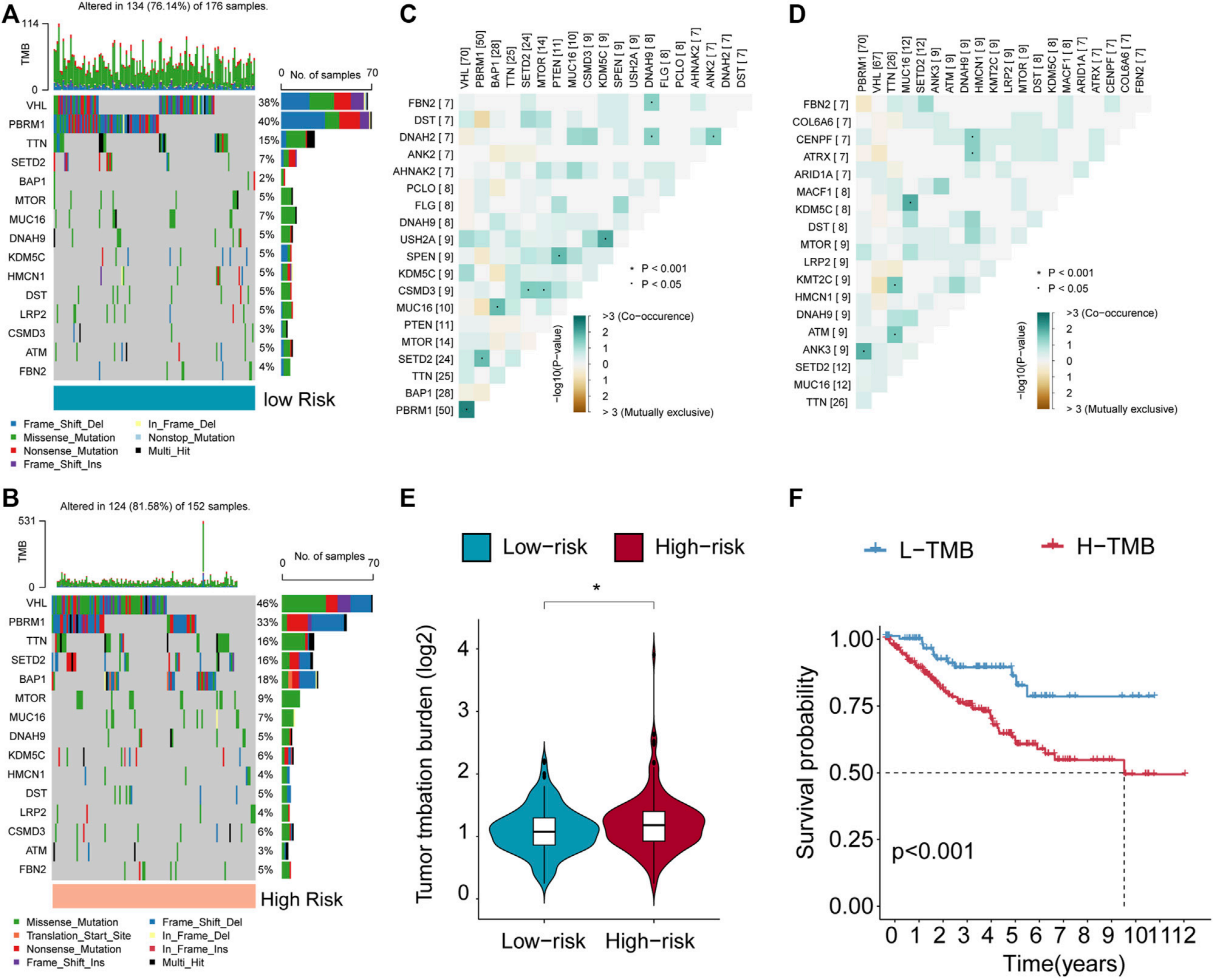
Features of VMRGS in tumor somatic mutations. **(A, B)** The Tumor Mutation Burden (TMB) differs among patients in the low- and high-risk score subcategories. **(C, D)** The interplay among the 20 most commonly mutated genes in high- and low-risk subgroups. **(E)** Violin plot illustrating TMB differences in ccRCC patients between the two risk groups. **(F)** Survival curves for patients categorized by both TMB were generated using the Kaplan-Meier method. Significance levels: *p < 0.05, **p < 0.01, ***p < 0.001.

The group at high risk had a record of the top 10 genes that underwent frequent mutations, which included VHL, PBRM1, TTN, SETD2, BAP1, MTOR, MUC16, DNAH9, KDM5C, and DST (Figure 6A). In contrast, the low-risk group also exhibited these genes as part of the top 10 most commonly mutated ones (Figure 6B). Their interactions are depicted in Figures 6C, D. The examination showed that ccRCC patients at high risk had a greater tumor mutation load (TMB) linked to reduced OS (Figures 6E, F). The results align with previous findings derived from Kaplan-Meier survival plots for both the high-risk and low-risk categories.

### Evaluation of signature gene expression levels and functions

**FIGURE 7:**
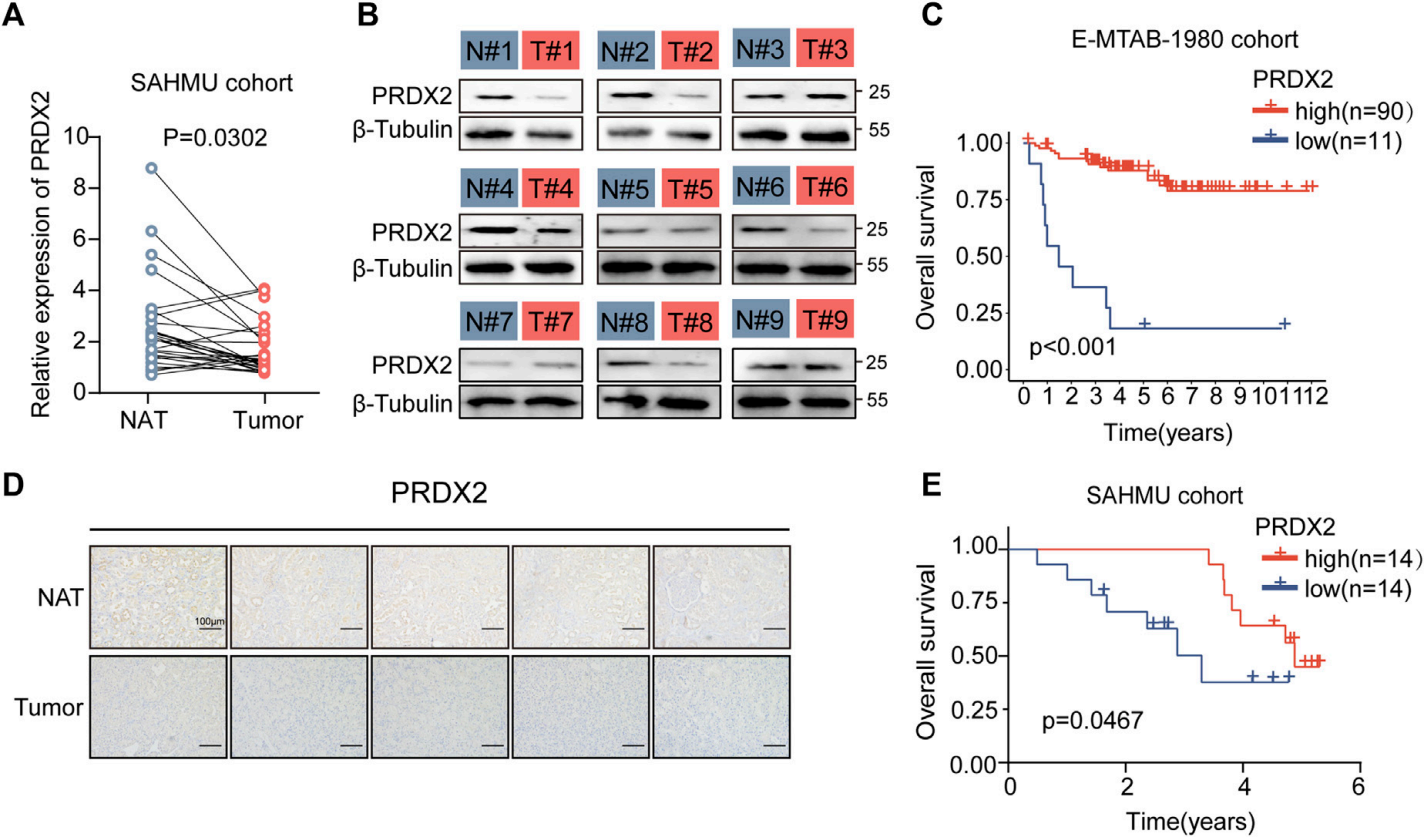
Low expression of PRDX2 in tumors is associated with poor prognosis. **(A)** Evaluation of PRDX2 mRNA levels in tumors (T) and corresponding normal adjacent tissues (NAT) within the SAHMU cohort (RT-qPCR data). **(B)** Western blot analysis depicting PRDX2 protein expression in nine pairs of human ccRCC tumors. **(C)** Survival curve (overall survival) based on distinct PRDX2 protein levels in the two ccRCC subtypes within the E-MTAB-1980 cohort. **(D)** Immunohistochemical images illustrating PRDX2 expression in tumors and corresponding normal adjacent tissues. Scale bar, 100 μm. **(E)** Survival curve (overall survival) based on different PRDX2 protein levels in the two ccRCC subtypes within the SAHMU cohort. Significance levels: *p < 0.05, **p < 0.01, ***p < 0.001.

In the cohort studies conducted at The Second Affiliated Hospital of Harbin Medical University (SAHMU), It was noted that the expression level of PRDX2 mRNA exhibited a substantial increase in ccRCC tissues. When compared to normal adjacent tissues (NAT) (n = 14 for normal, n = 14 for ccRCC) (Figure 7A). Immunohistochemistry staining (Figure 7D) and Western blot analyses (Figure 7B) confirmed the reduced expression of PRDX2 in paired ccRCC tissue samples. In both the E-MTAB-1980 cohort (Figure 7C) and the SAHMU cohort (Figure 7E), the Kaplan-Meier survival curves indicated that a decreased PRDX2 expression level in ccRCC patients correlated with unfavorable OS. To summarize, our results offer corroborating proof for the reduced PRDX2 expression in ccRCC, emphasizing its negative correlation with the prognostic results of ccRCC individuals.

Additionally, we corroborated the influence of PRDX2 on the formation of VM. Following the knockdown of PRDX2 (Supplementary Figure S4A), there was a marked decrease in VM formation (Supplementary Figure S4B). This suggests that PRDX2 actively encourages the development of VM.

### Disscusion

A large proportion of RCC occur as ccRCCs in humans. Due to substantial advancements in the clinical management of ccRCC, a comprehensive understanding of various prognostic factors such as tumor grade, tumor stage, and tumor size has been achieved. Additionally, alterations in genes and molecules are observed in ccRCC (Linehan et al., 2016). Several biological processes may be impacted, and certain among them are intricately linked to a patient’s prognosis for ccRCC., such as autophagy (Napolitano et al., 2020), ferroptosis (Miess et al., 2018) and redox (Qi-Dong et al., 2020).

ccRCC has been revolutionized by immunotherapies that target co-inhibitory immune checkpoints, resulting in the emergence of immune-checkpoint inhibitors (Rathmell et al., 2022). Nonetheless, after a brief but effective treatment regimen, some patients experience unresponsiveness or secondary drug resistance, subsequently leading to disease progression (Jenkins et al., 2018; Schoenfeld and Hellmann et al., 2020). Since no widely accepted signature exists for predicting immunotherapy sensitivity in ccRCC, A dependable bioindicator for forecasting the susceptibility to immunotherapeutic agents needs to be ascertained.

VM, a phenomenon that promotes an augmentation in blood supply, Plays an indispensable role in the formation of solid neoplasms. It is imperative to form new blood vessels when the tumor diameter exceeds 2 mm to maintain adequate oxygenation. Failure to do so may result in ischemia and hypoxia, leading to necrosis of the tumor (Folkman, 1971; Zhang et al., 2022).

Given the limited efficacy of anti-angiogenic therapies in suppressing tumor development, it is imperative to explore innovative strategies for combating tumor angiogenesis that focus on targeting alternative mechanisms employed by tumor cells, specifically trans-differentiation, which enables them to assume a pseudo-vascular phenotype and promote VM (Delgado-Bellido et al., 2017).

Angiogenesis significantly influences ccRCC development. The procedure includes a intricate web of signaling cascades comprising of elements such as pVHL, HIF-1α, VEGF, PDGF, and mTOR (Nicol et al., 1997; Dorević et al., 2009; Dimova et al., 2014; Kornakiewicz et al., 2014). Currently, approved targeted therapy agents for progressed ccRCC include bevacizumab, a monoclonal antibody that blocks VEGF-A from binding to its receptor. Furthermore, there are TKIs such as sorafenib, sunitinib, pazopanib, and axitinib, which predominantly hinder the VEGF receptor. Furthermore, mTOR complex inhibitors like temsirolimus and everolimus are also used. It is worth noting that immunocompromised patients face an increased risk of ccRCC, tumors often contain abundant lymphocytes, and occasional spontaneous tumor regressions have been reported (McDermott and Rini, 2007; Di Lorenzo et al., 2010; Mickley et al., 2015; Cecere et al., 2016; Di Lorenzo et al., 2016). The field of immunotherapy for ccRCC has witnessed a noteworthy progression with the deployment of agents that aim to regulate immune suppression induced by tumors. Particularly, the utilization of nivolumab, which targets PD-1, and ipilimumab, which targets CTLA-4, has exhibited encouraging outcomes.

Through an extensive examination of available literature, a total of 43 VMRGs were initially acquired in this investigation. Subsequently, individuals with ccRCC were randomly allocated to either the training or test group. Following the implementation of univariate Cox regression to detect 20 prognostic indicators for DE-VMRG, LASSO Cox regression was employed to identify four distinct VMRG signatures. Patients with high-risk scores had a more unfavorable prognosis in the KM survival analysis, in contrast to patients with low-risk ccRCC. The newly discovered signature demonstrated strong prognostic potential. Moreover, individuals diagnosed with ccRCC demonstrated consistent outcomes across various risk categories based on an evaluation of the area under the curve (AUC). Hence, evaluating the levels of VMRG expression can function as an indicator of death risk in ccRCC individuals, by utilizing the expression levels of four VMRGs. Altered expressions of MMPs and their inhibitors, TIMPs, have been observed in various tumor tissues. In particular, MMP14 exhibited significantly greater levels of expression in clear cell and papillary RCCs when compared to non-cancerous tissue, which generally had low MMP levels.

Many of the genes in the risk signature have a significant impact on the control of cancer due to their crucial functions. Through lncRNA-TANAR, the Androgen receptor modulates TWIST1 nonsense-mediated decay in ccRCC to induce VM. Targeting the AR/TANAR/TWIST1 signaling pathways with a novel anti-angiogenesis treatment shows promise in halting the advancement of ccRCC (You et al., 2021). Cancer researchers have established that KDR is an important clinical biomarker and a key drug target in numerous solid tumors. Our signature, however, shows that KDR plays a protective role, which is unexpected. Further investigation is necessary for this observation, especially considering the positive outcomes achieved by different inhibitors of VEGF receptors in improving the prognosis of ccRCC.

Peroxiredoxin 2 (PRDX2) is a member of the peroxiredoxin family and protects cells from oxidative stress by scavenging ROS and H2O2 (De Franceschi et al., 2011). It has been shown that PRDX2 can suppress or enhance tumorigenesis depending on context, cells, and cancer type, etiology, and stage (Lei et al., 2016; Nicolussi et al., 2017). Proliferation and migration are promoted by melanoma PRDX2 expression, which is also linked to EMT and β-catenin signaling (Furuta et al., 2006). Meanwhile, PRDX2 overexpression correlates with cancer progression in several malignancies, including colon, prostate, cervix, and lung (Lomnytska et al., 2011; Xu et al., 2017). As a result of excessive levels of oxidative molecules, a knockdown of peroxiredoxin-2 reduced VEGFR-2 activation in colorectal cancer and caused VM formation (Zhang et al., 2015a). According to the findings from real-time quantitative PCR and immunohistochemistry, it was observed that PRDX2 expression in normal renal cells was considerably higher in comparison to ccRCC cell lines.

Additionally, in our study, we conducted validation of the impact of PRDX2 on VM formation in ccRCC, which is similar to its effect in colorectal cancer (Zhang et al., 2015b), demonstrating a promotion effect. In our previous studies, we observed a positive correlation between VM and poor prognosis in renal cancer (Liu et al., 2022). However, it is noteworthy that PRDX2 exhibits a negative correlation with adverse outcomes in ccRCC. Consequently, whether VM exerts a favorable influence on renal cancer prognosis through alternative pathways or if PRDX2 affects ccRCC prognosis via mechanisms distinct from VM remains unclear. We intend to further explore these mechanisms in our subsequent research endeavors.

The development and utilization of immune-checkpoint inhibitors that target CTLA4, PD-1, and PD-L1 have had a transformative impact on the field of cancer therapy, as acknowledged by the 2018 Nobel Prize in Medicine and Physiology (Braun et al., 2021; Kraehenbuehl et al., 2022), This has introduced a novel immunotherapy strategy with promising potential for treating cancer. Based on the positive outcomes from the randomized phase III trial CheckMate-025 (CM-025), the FDA has granted approval for the use of nivolumab (anti-PD-1) in the management of ccRCC. In prior studies, nivolumab exhibited enhanced OS when compared to everolimus, an mTOR inhibitor, in patients with previously treated ccRCC (Cancer Genome Atlas Research Network, 2013; Miao et al., 2018; Braun et al., 2020).

The study revealed that the group at high risk exhibited elevated levels of expression for the majority of checkpoint indicators, particularly PD-1. These results have important implications for identifying ccRCC patients likely to benefit from ICI therapy.

Additionally, we substantiated the reduced PRDX2 expression within ccRCC tissues through an in-depth examination of both patient mRNA and protein expression profiles. Furthermore, we corroborated its adverse correlation with patient prognosis.

## Conclusion

The identified and validated four-VMRG signature may function as a valuable biomarker for ccRCC, offering a potential strategy for treatment. This research may enable us to predict prognosis and formulate efficient chemotherapy and immunotherapy for ccRCC patients.

## Data Availability

Publicly available datasets were analyzed in this study. This data can be found here: The datasets used in this article were derived from the TCGA database (https://portal.gdc.cancer.gov/), the GEO database (https://www.ncbi.nlm.nih.gov/geo/), the accession number is GSE29609) and the EMBL-EBI database (https://www.ebi.ac.uk/biostudies/arrayexpress/studies/, the accession number involves E-MTAB-1980).
