# Supplementary material for: The categorizations of vasculogenic mimicry in clear cell renal cell carcinoma unveil inherent connections with clinical and immune features
Source: Front Pharmacol. 2023 Dec 20;14:1333507. doi: 10.3389/fphar.2023.1333507 (PMC10765515; doi:10.3389/fphar.2023.1333507)
Supplement: Supplementary file 1 [file Table1.DOCX]

| id | coef |
| --- | --- |
| TWIST1 | 0.431593600306808 |
| PRDX2 | -0.202507127424664 |
| KDR | -0.381746728029345 |
| MMP14 | 0.313954576782016 |
