# Supplementary figures and images for: The categorizations of vasculogenic mimicry in clear cell renal cell carcinoma unveil inherent connections with clinical and immune features
Source: Front Pharmacol. 2023 Dec 20;14:1333507. doi: 10.3389/fphar.2023.1333507 (PMC10765515; doi:10.3389/fphar.2023.1333507)

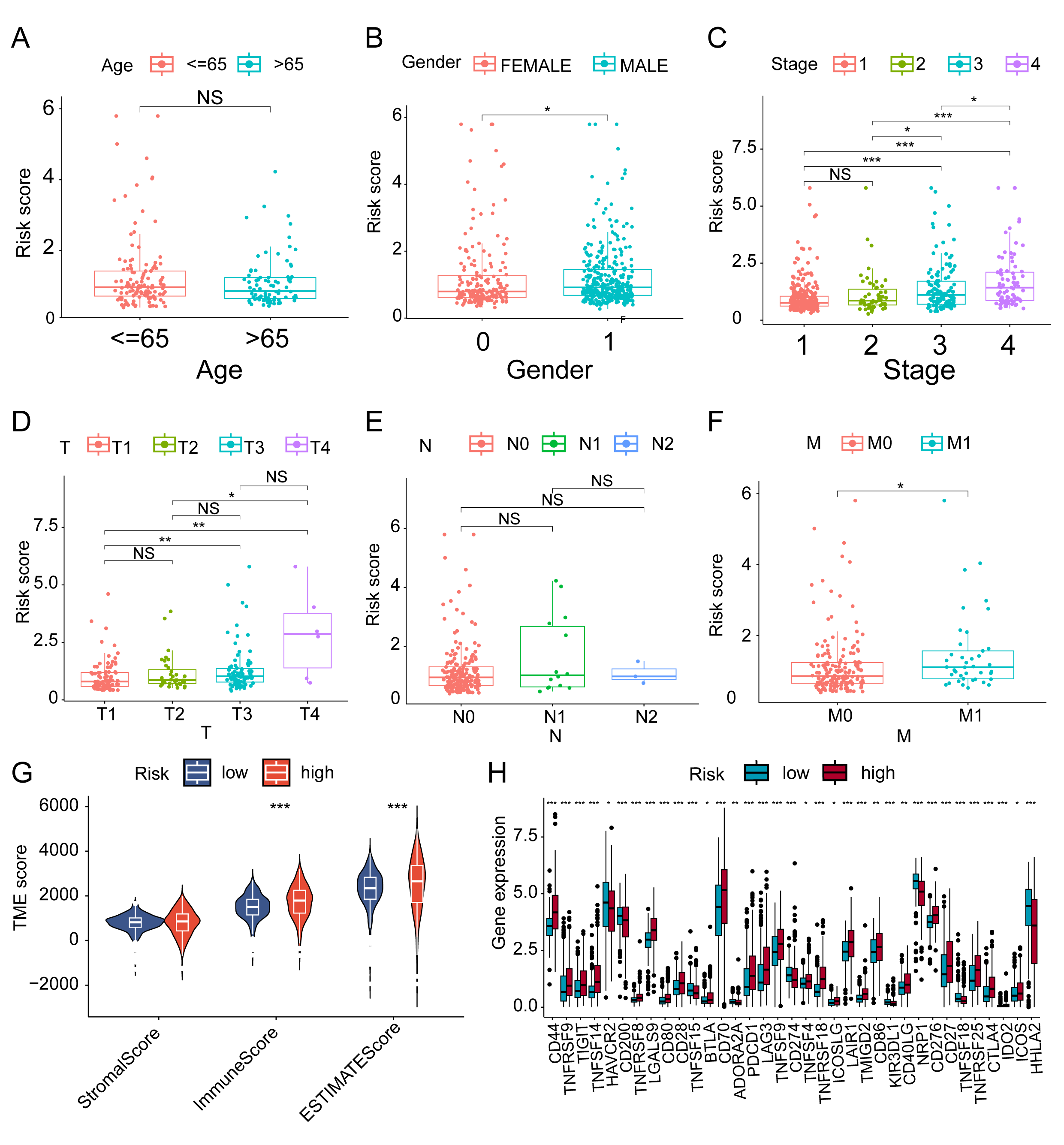

Supplement: Supplementary file 2 [file Image3.TIF]

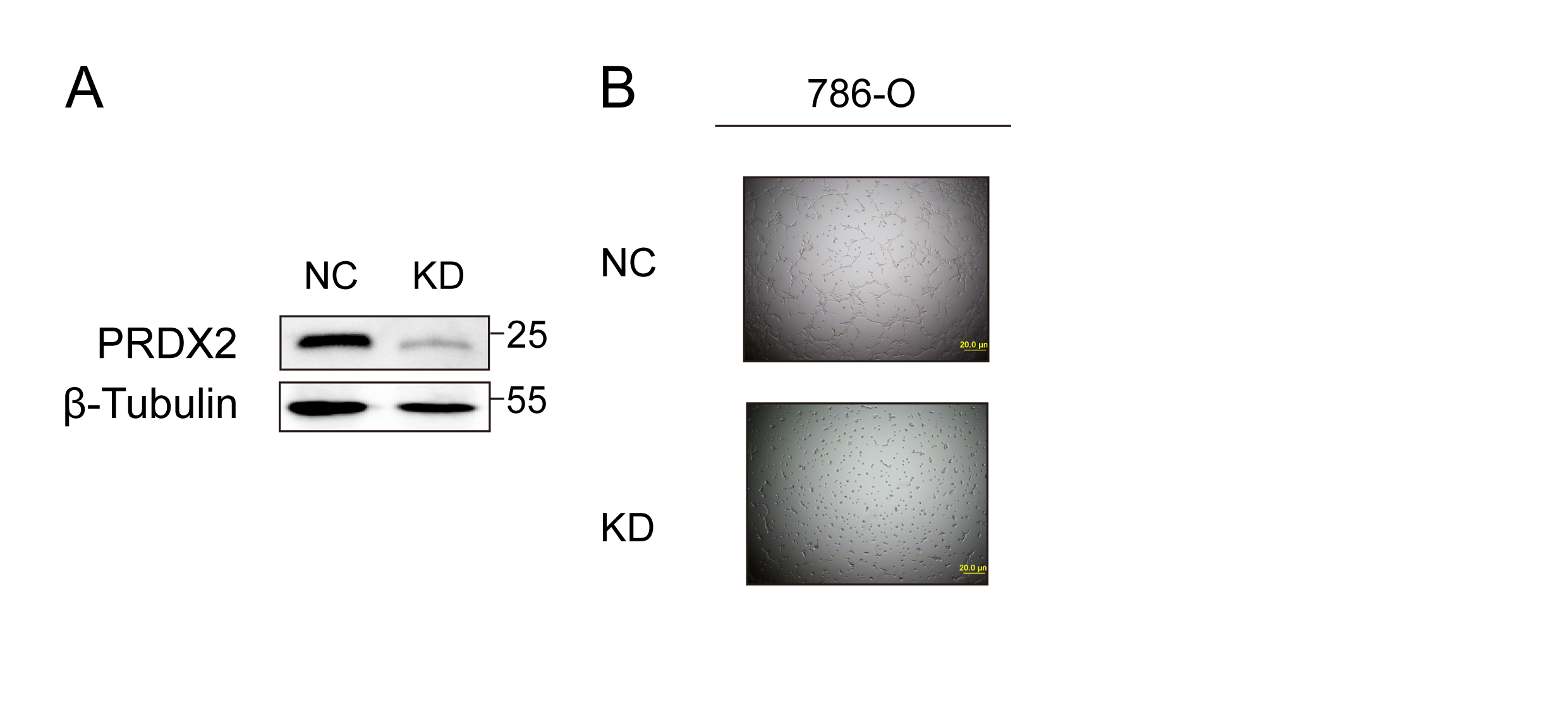

Supplement: Supplementary file 3 [file Image4.TIF]

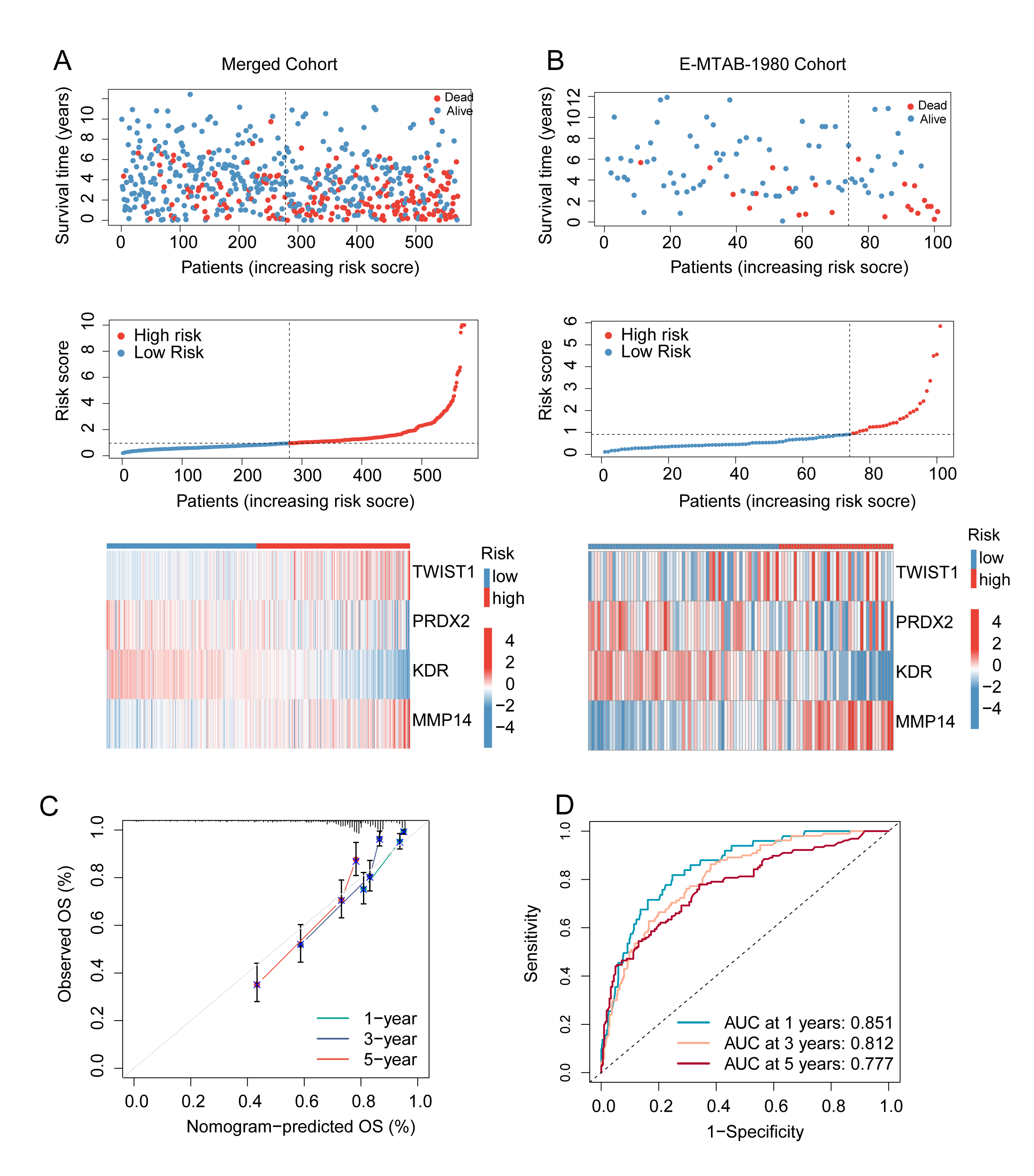

Supplement: Supplementary file 4 [file Image2.TIF]

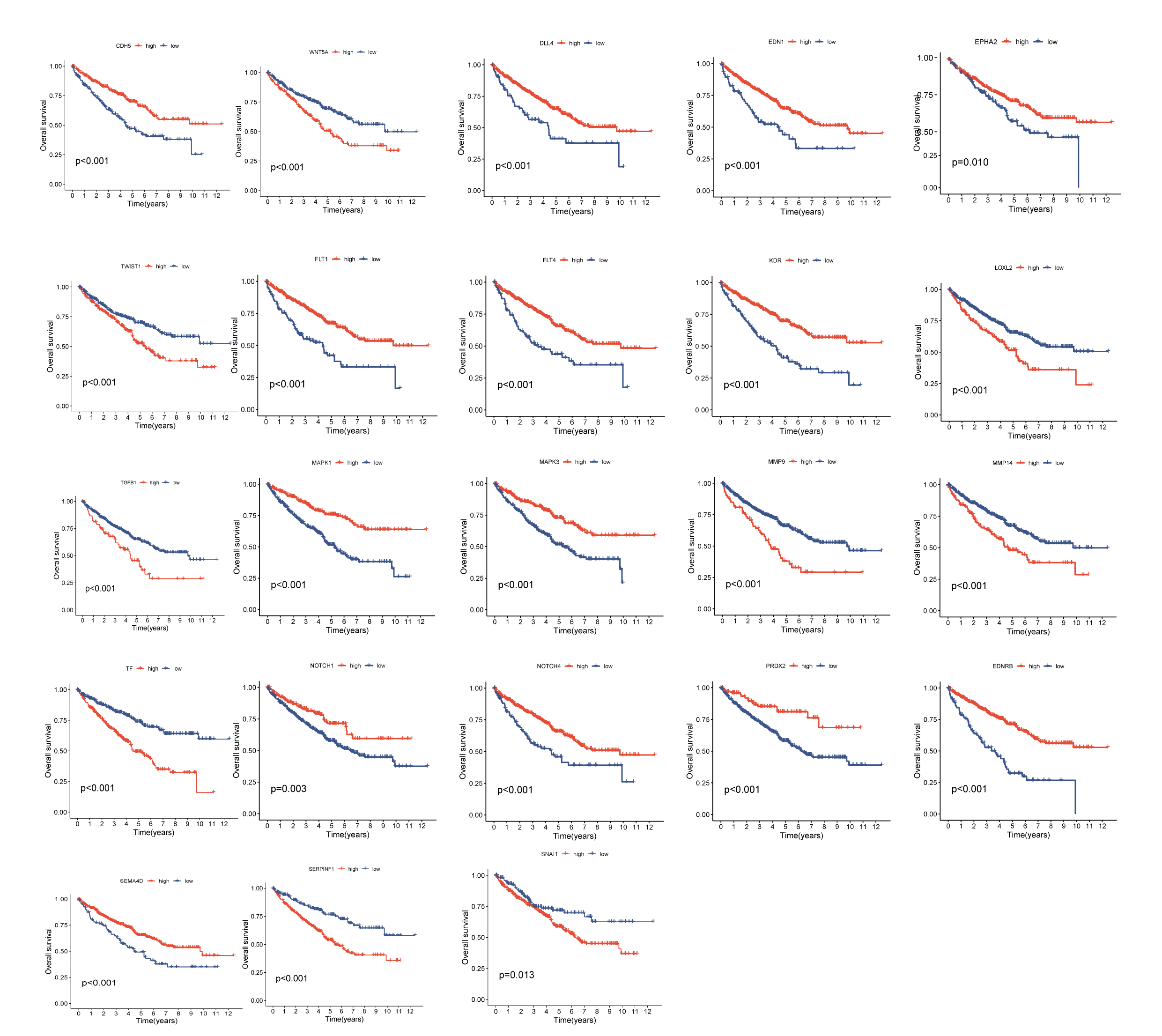

Supplement: Supplementary file 5 [file Image1.TIF]
