# Supplementary material for: The categorizations of vasculogenic mimicry in clear cell renal cell carcinoma unveil inherent connections with clinical and immune features
Source: Front Pharmacol. 2023 Dec 20;14:1333507. doi: 10.3389/fphar.2023.1333507 (PMC10765515; doi:10.3389/fphar.2023.1333507)
Supplement: Supplementary file 6 [file Table2.DOCX]

| **Table S2** The sequences of primers used in this study. | |
| --- | --- |
| PRDX2-F | CCACCTGGCTTGGATCAACA |
| PRDX2-R | TTTCAGCACGCCGTAATCCT |
